# Bacteria richness and antibiotic-resistance in bats from a protected area in the Atlantic Forest of Southeastern Brazil

**DOI:** 10.1371/journal.pone.0203411

**Published:** 2018-09-14

**Authors:** Vinícius C. Cláudio, Irys Gonzalez, Gedimar Barbosa, Vlamir Rocha, Ricardo Moratelli, Fabrício Rassy

**Affiliations:** 1 Centro de Ciências Biológicas e da Saúde, Universidade Federal de São Carlos, São Carlos, SP, Brazil; 2 Fundação Parque Zoológico de São Paulo, São Paulo, SP, Brazil; 3 Instituto de Biologia, Universidade Federal do Rio de Janeiro, Rio de Janeiro, RJ, Brazil; 4 Centro de Ciências Agrárias, Universidade Federal de São Carlos, Araras, SP, Brazil; 5 Fiocruz Mata Atlântica, Fundação Oswaldo Cruz, Rio de Janeiro, RJ, Brazil; Institut National de la Recherche Agronomique, FRANCE

## Abstract

Bats play key ecological roles, also hosting many zoonotic pathogens. Neotropical bat microbiota is still poorly known. We speculate that their dietary habits strongly influence their microbiota richness and antibiotic-resistance patterns, which represent growing and serious public health and environmental issue. Here we describe the aerobic microbiota richness of bats from an Atlantic Forest remnant in Southeastern Brazil, and the antibiotic-resistance patterns of bacteria of clinical importance. Oral and rectal cavities of 113 bats from Carlos Botelho State Park were swabbed. Samples were plated on 5% sheep blood and MacConkey agar and identified by the MALDI-TOF technique. Antibiotic susceptibility tests were performed using Kirby-Bauer’s antibiotic disc diffusion technique.We identified 596 isolates at the genus level and tentatively to the species level. Proteobacteria was the most abundant phylum in all the dietary guilds, representing 87% of the total identified samples. The most common bacteria within bat individuals were *Escherichia coli*, *Klebsiella oxytoca* and *Serratia marcescens*, and within bat species were *Serratia marcescens*, *Pseudomonas* sp. and *Staphylococcus* sp. Frugivores presented the most diverse microbiota. In general, the antibiogram results indicated a low occurrence of resistance on eigth potentially pathogenic bacteria species. The resistance to antibiotics found on our samples was related mostly to the intrinsic resistance of the tested species.The low occurrence of resistant bacteria in our samples could be related to the well preserved environment where bats were caught. Once the major causes of resistance-acquiring are related to anthropic activites, the controlled access of tourists on certain regions of the Park seems to be effectively protecting the environment.

## Introduction

Bats as a group are distributed worldwide, with more than 1300 species, representing ca. 20% of the world mammals [1]. They are highly diversified ecologically, bringing together the most diversified feeding strategies among terrestrial vertebrates. Dietary strategies include frugivory, hematophagy, insectivory, nectarivory, carnivory, piscivory and omnivory [1, 2]. Some species allocated in one of these categories include different food sources in their diet [1, 2]. Due to this diversified diet they provide important ecosystem services such as seed dispersal, pollination and pest control, but also carry many pathogens, some of them of zoonotic potential [3, 4]. Little is known about Neotropical bat microbiota, which is in great part studied for Old World species and mostly related to the gastrointestinal diversity [5–11];. Also, studies focused on the interaction, influence and ecologic role of bats oral and rectal microbiota are scarce, despite their importance on the digestion, vitamin synthesis, protection against harmful microorganisms and also public health [12–17].

Previous studies of bat gut microbiota showed that the bacteria diversity is in part related to the host diet, with a partial overlap between species in different dietary guilds, once these species can compensate the lack of some requirements with different food sources during resources shortages [9, 18]. Besides the microbiota diversity, the bacteria antibiotic-resistance patterns could be also modulated by dietary habits [19–21]. Among the major causes of resistance acquiring is the contact with anthropic environments [21, 22]. Antimicrobial resistant bacteria are a growing and serious problem to the public health and environment, and are reported to be present even on remote habitats [17, 23]. The presence of antimicrobial resistance in wildlife brings implications, as it can drive animals to become potential reservoirs of resistant bacteria, and also impose limits to the efficiency of antibiotics used on the control of human and wildlife diseases [17, 21].

Against that background, we aimed (1) to describe the oral and rectal aerobic microbiota richness of bats in five dietary guilds from the Carlos Botelho State Park (CBSP), a protected area on the Atlantic Forest of Southeastern Brazil, focusing bacteria of clinical importance; (2) to identify the antibiotic-resistance profile of eight potentially pathogenic bacteria for those bats; and (3) to evaluate whether the protected area is preserving the wildlife from antibiotic resistant bacteria.

## Material and methods

### Sampling

Fieldwork was conducted monthly from October 2016 to September 2017 on the Carlos Botelho State Park (CBSP; 24°12'–24°4'S, 47°47'–48°7'W), which is a protected area in the Brazilian Southeastern Atlantic Forest, created in 1982. The phytophysiognomy is mostly represented by the ombrophilous forest, with ca. 23,300 ha composed by pristine forests [24]. Bats were captured using with mist-nets and during searches for roosts, under the permits SISBIO/ICMBIO 54.381-1/2016 and COTEC/SMA-IF 260108006.479/2016. Monthly, oral and rectal cavities of one bat of each species captured were swabbed with sterile cotton swabs, which were then separately transported in Stuart’s transport medium and refrigerated. Samples used in this study were collected from 113 bats of 33 species, divided into five dietary guilds (frugivores [FRU]; insectivores [INS]; nectarivores [NEC]; sanguivores [SAN]; and carnivores [CAR]).

### Isolation and identification of the microbiota

Samples collected in fieldwork were plated on 5% sheep blood agar and MacConkey agar, and incubated aerobically at 36°C for 24h. Colonies were further isolated by morphotype and preserved in Tryptic Soy Broth and 20% glycerol at -80°C; all the isolates are stocked at the Culture Collection of the Fundação Parque Zoológico de São Paulo. The isolates were later identified by the matrix-assisted laser desorption/ionization (MALDI) technique, using MALDI Biotyper System in collaboration with the Proteomics Laboratory at Universidade Federal de São Paulo [25]. The database of this technique is mostly composed by pathogenic species, therefore a great part of the identifications tend to result on pathogenic bacteria species. Isolates were analyzed using a formic acid-based direct, on-plate preparation method. Small amounts of a single colony were smeared directly onto a spot of the MALDI-TOF MS steel anchor plate. Each spot was then overlaid with one microliter of 70% formic acid and allowed to dry. The dried mixture was overlain with 1 μl of matrix solution (α-cyano-4-hydroxycinnamic acid [HCCA]) dissolved in 50% acetonitrile, 47.5% water, and 2.5% trifluoroacetic acid and allowed to dry prior to analysis using the MALDI Biotyper. An *Escherichia coli* (ATCC 25922) isolate was used for instrument calibration. Two positive controls (*Escherichia coli* ATCC 25922 and *Staphylococcus aureus* ATCC 25923) were included with each run [26].

### Antibiotic sensitivity

Antibiotic susceptibility tests were performed on Mueller Hinton agar using Kirby-Bauer’s antibiotic disc diffusion technique [27]. The tests were performed for the most potentially pathogenic bacteria species *Acinetobacter baumannii*, *Escherichia coli*, *Klebsiella oxytoca*, *Pseudomonas aeruginosa*, *Salmonella* sp., *Serratia marcescens*, *Stenotrophomonas maltophilia*, and *Stenotrophomonas* sp. The antibiotics used on the tests were selected according to the bacteria characteristics [28], and the discs were firmly placed on the seeded plates, which were incubated at 36°C for 24h. The susceptibility of each isolate for different antibiotics was evaluated by the zones of inhibition, which were measured and compared with the susceptibility pattern of each antibiotic defined by the Clinical and Laboratory Standards Institute [28].

The antibiotics tested for *Acinetobacter baumannii* were: amikacin (AMI, 30 μg), ceftazidime (CAZ, 30 μg), ceftriaxone (CRO, 30 μg), ciprofloxacin (CIP, 5 μg), chloramphenicol (CLO, 30 μg), gentamicin (GEN, 10 μg), imipenem (IPM, 10 μg) and norfloxacin (NOR, 10 μg). The antibiotics tested for *Pseudomonas aeruginosa* were: ceftazidime (CAZ, 30 μg), ceftriaxone (CRO, 30 μg), ciprofloxacin (CIP, 5 μg), gentamicin (GEN, 10 μg), imipenem (IPM, 10 μg) and norfloxacin (NOR, 10 μg). The antibiotics tested for *Stenotrophomonas maltophilia* and *Stenotrophomonas* sp. were: ceftazidime (CAZ, 30 μg), ceftriaxone (CRO, 30 μg), ciprofloxacin (CIP, 5 μg), gentamicin (GEN, 10 μg), imipenem (IPM, 10 μg), norfloxacin (NOR, 10 μg) and trimethoprim-sulphamethoxazole (SUT, 1.25/23.75 μg). The antibiotics tested for *Escherichia coli*, *Klebsiella oxytoca*, *Salmonella* sp. and *Serratia marcescens* were: amikacin (AMI, 30 μg), ceftazidime (CAZ, 30 μg), ceftriaxone (CRO, 30 μg), ciprofloxacin (CIP, 5 μg), chloramphenicol (CLO, 30 μg), gentamicin (GEN, 10 μg), imipenem (IPM, 10 μg), doxycycline (DOX, 30 μg), ampicillin (AMP, 10 μg), amoxicillin-clavulanate (AMC, 20/10 μg) and cephalexin (CFL, 30 μg).

## Results

### Oral and rectal microbiota

We isolated 830 morphotypes of bacteria from bats in five different dietary guilds (carnivores, frugivores, insectivores, nectarivores and sanguivores). A total of 596 morphotypes were identified at the genus level and tentatively to the species level by the MALDI-TOF methodology, including 243 from the oral cavity and 353 from the rectal cavity. Successfully identified isolates from the oral cavity are represented by: 14 isolates from two species of carnivores; 15 isolates from two species of sanguivores; 25 isolates from three species of nectarivores; 75 isolates from 14 species of insectivores; and 113 isolates from 10 species of frugivores (Table 1). Successfully identified isolates from the rectal cavity are represented by: 11 isolates from two species of carnivores; 27 isolates from two species of sanguivores; 60 isolates from two species of nectarivores; 90 isolates from 15 species of insectivores; and 165 isolates from 11 species of frugivores (Table 2).

**Table 1:** Successfully identified oral microbiota from bats of Carlos Botelho State Park, São Paulo State.

Species (Number of specimens)			Diet	Oral Microbiota (Number of isolates)
**Family Phyllostomidae**				
	**Subfamily Micronycterinae**			
		*Micronycteris microtis*(2)	*Insectivore*	*Hafnia alvei* (2); *Serratia marcescens* (1); *Streptococcus gallinaceus* (1)
		*Micronycteris schimdtorum*(1)	*Insectivore*	*-*
	**Subfamily Desmodontinae**			
		*Desmodus rotundus*(6)	*Sanguivore*	*Acinetobacter* sp. (1); *Arthrobacter* sp. (1); *Klebsiella* sp. (1); *Kluyvera* sp. (1); *Pantoea* sp. (1); *Pseudomonas stutzeri* (2); *Raoultella* sp. (1); *Serratia marcescens* (2); *Serratia* sp. (1); *Staphylococcus aureus* (1); *Streptococcus gallinaceus* (1)
		*Diphylla ecaudata*(3)	*Sanguivore*	*Staphylococcus* sp. (2)
	**Subfamily Phyllostominae**			
		*Mimon bennetti*(1)	*Carnivore*	*Citrobacter freundii* (1); *Enterobacter* sp. (2); *Klebsiella* sp. (1); *Lactococcus lactis* (1); *Serratia marcescens* (1)
		*Trachops cirrhosus*(2)	*Carnivore*	*Aeromonas hydrophila* (2); *Kluyvera ascorbata* (2); *Lactococcus lactis* (1); *Serratia marcescens* (3)
	**Subfamily Glossophaginae**			
		*Anoura caudifer*(9)	*Nectarivore*	*Arthrobacter* sp.(1); *Cedecea lapagei* (1); *Lactococcus lactis* (1); *Microbacterium* sp. (1); *Pseudomonas fulva* (1); *Pseudomonas koreensis* (1); *Pseudomonas* sp.(1); *Rahnella* sp. (2); *Serratia marcescens* (6); *Staphylococcus aureus* (1); *Streptococcus* sp. (1)
		*Anoura geoffroyi*(7)	*Nectarivore*	*Arthrobacter* sp. (1); *Enterobacter cloacae* (1); *Pantoea agglomerans* (1); *Pantoea* sp. (1); *Pseudomonas* sp. (1); *Staphylococcus aureus* (1); *Staphylococcus* sp. (1)
		*Glossophaga soricina*(1)	*Nectarivore*	*Staphylococcus* sp. (1)
	**Subfamily Carolliinae**			
		*Carollia perspicillata*(8)	*Frugivore*	*Escherichia* sp. (1); *Escherichia vulneris* (1); *Neisseria* sp. (1); *Pseudomonas aeruginosa* (1); *Pseudomonas extremorientalis* (2); *Pseudomonas* sp. (1); *Serratia liquefaciens* (1); *Serratia marcescens* (5); *Staphylococcus* sp. (1); *Stenotrophomonas maltophilia* (1)
	**Subfamily Glyphonycterinae**			
		*Glyphonycteris sylvestris*(2)	*Insectivore*	*Serratia* sp. (4)
	**Subfamily Stenodermatinae**			
		*Artibeus fimbriatus*(9)	*Frugivore*	*Acinetobacter* sp. (1); *Arthrobacter* sp. (1); *Burkholderia* sp. (1);*Enterobacter cloacae* (2); *Enterobacter* sp. (3); *Klebsiella oxytoca* (2); *Pseudomonas* sp. (3); *Raoultella ornithinolytica* (1); *Raoultella terrigena* (4); *Serratia marcescens* (3); *Serratia* sp. (2); *Stenotrophomonas* sp.(1)
		*Artibeus lituratus*(5)	*Frugivore*	*Acinetobacter* sp. (2); *Lactococcus* sp. (1); *Leclercia adecarboxylata* (1); *Leclercia* sp. (1); *Pantoea agglomerans* (1); *Pantoea* sp. (1); *Salmonella* sp. (1); *Serratia marcescens* (2); *Serratia* sp. (1); *Staphylococcus saprophyticus* (1); *Streptococcus* sp. (1)
		*Artibeus obscurus*(8)	*Frugivore*	*Enterobacter* sp. (1); *Leclercia adecarboxylata* (1); *Ochrobactrum intermedium* (1); *Ochrobactrum* sp. (1); *Pantoea agglomerans* (6); *Pseudomonas koreensis* (2); *Pseudomonas* sp. (6); *Serratia marcescens* (5); *Serratia* sp. (1); *Stenotrophomonas* sp. (1)
		*Dermanura cinerea*(2)	*Frugivore*	*Pantoea* sp. (3); *Serratia marcescens* (1); *Serratia* sp.(2)
		*Platyrrhinus lineatus*(1)	*Frugivore*	*Enterobacter asburiae* (1); *Klebsiella oxytoca* (1); *Klebsiella* sp. (1)
		*Platyrrhinus recifinus*(1)	*Frugivore*	*Enterobacter* sp. (2); *Serratia marcescens* (2)
		*Pygoderma bilabiatum*(2)	*Frugivore*	*-*
		*Sturnira lilium*(8)	*Frugivore*	*Acinetobacter lwoffii* (1); *Escherichia coli* (2); *Hafnia* sp. (2); *Lactococcus lactis* (1); *Pantoea agglomerans* (1); *Pantoea ananatis* (2); *Pseudomonas* sp. (3); *Streptococcus* sp. (1)
		*Sturnira tildae*(5)	*Frugivore*	*Acinetobacter* sp. (1); *Bacillus thuringiensis* (1); *Enterobacter* sp. (1); *Escherichia coli* (2); *Leclercia* sp. (1); *Pseudomonas* sp. (1); *Stenotrophomonas* sp. (1)
		*Vampyressa pusilla*(1)	*Frugivore*	*Enterobacter* sp. (2)
**Family Molossidae**				
	**Subfamily Molossinae**			
		*Cynomops abrasus*(1)	*Insectivore*	*Acinetobacter pittii* (2); *Enterobacter cloacae* (1)
		*Molossops neglectus*(1)	*Insectivore*	*Enterobacter* sp. (1); *Escherichia coli* (1); *Serratia marcescens* (2); *Staphylococcus* sp. (1)
		*Molossus currentium*(1)	*Insectivore*	*Hafnia* sp. (1); *Serratia marcescens* (4)
		*Molossus molossus*(4)	*Insectivore*	*Cedecea lapagei* (1); *Citrobacter* sp. (1); *Ochrobactrum* sp. (1); *Ochrobactrum tritici* (1); *Serratia marcescens* (2); *Serratia* sp. (1); *Staphylococcus* sp. (1)
		*Molossus rufus*(2)	*Insectivore*	*Acinetobacter baumannii* (1); *Acinetobacter* sp. (1); *Escherichia coli* (1); *Proteus vulgaris* (1); *Salmonella* sp. (1); *Serratia marcescens* (2)
**Family Vespertilionidae**				
	**Subfamily Vespertilioninae**			
		*Eptesicus taddeii*(1)	*Insectivore*	*Serratia* sp. (2)
		*Lasiurus ebenus*(1)	*Insectivore*	*Enterobacter cloacae* (1); *Pseudomonas aeruginosa* (2); *Serratia marcescens* (3)
		*Histiotus velatus*(3)	*Insectivore*	*Enterobacter* sp. (1); *Erwinia persicina* (1); *Hafnia alvei* (2); *Pseudomonas* sp.(1); *Serratia marcescens* (2); *Staphylococcus* sp. (1)
	**Subfamily Myotinae**			
		*Myotis albescens*(1)	*Insectivore*	*Staphylococcus* sp. (2)
		*Myotis nigricans*(6)	*Insectivore*	*Aeromonas hydrophila* (1); *Enterobacter* sp. (2); *Lactococcus lactis* (1); *Pantoea agglomerans* (1); *Pantoea* sp. (1); *Serratia marcescens* (3); *Serratia* sp. (2); *Yokenella regensburgei* (1)
		*Myotis riparius*(2)	*Insectivore*	*Serratia marcescens* (2)
		*Myotis ruber*(2)	*Insectivore*	*Enterococcus faecalis* (1); *Ewingella americana* (2); *Pseudomonas* sp. (1); *Serratia marcescens* (2); *Serratia* sp. (1)

**Table 2:** Successfully identified rectal microbiota from bats of Carlos Botelho State Park, São Paulo State.

Species (Number of specimens)			Diet	Rectal Microbiota (Number of isolates)
**Family Phyllostomidae**				
	**Subfamily Micronycterinae**			
		*Micronycteris microtis*(2)	*Insectivore*	*Citrobacter koseri* (1); *Citrobacter* sp. (1); *Enterobacter cloacae* (1); *Hafnia alvei* (2)
		*Micronycteris schimdtorum*(1)	*Insectivore*	*Staphylococcus* sp. (2)
	**Subfamily Desmodontinae**			
		*Desmodus rotundus*(6)	*Sanguivore*	*Acinetobacter* sp. (1); *Brevundimonas* sp. (1); *Citrobacter* sp. (2); *Edwardsiella* sp. (1); *Escherichia coli* (3); *Klebsiella oxytoca* (4); *Klebsiella* sp. (2); *Pantoea* sp. (2); *Pseudomonas* sp. (1); *Staphylococcus aureus* (1); *Staphylococcus* sp. (1)
		*Diphylla ecaudata*(3)	*Sanguivore*	*Enterobacter cloacae* (1); *Enterobacter* sp. (1); *Escherichia* sp. (3); *Klebsiella oxytoca* (1); *Serratia marcescens* (2)
	**Subfamily Phyllostominae**			
		*Mimon bennetti*(1)	*Carnivore*	*Citrobacter freundii* (1); *Hafnia alvei* (1); *Klebsiella oxytoca* (1); *Kluyvera ascorbata* (1); *Vagococcus fluvialis* (1)
		*Trachops cirrhosus*(2)	*Carnivore*	*Escherichia coli* (2); *Kluyvera ascorbata* (3); *Serratia marcescens* (1)
	**Subfamily Glossophaginae**			
		*Anoura caudifer*(9)	*Nectarivore*	*Acinetobacter baylyi* (1); *Acinetobacter* sp. (2); *Bacillus* sp. (2); *Cedecea lapagei* (3); *Enterobacter radicincitans* (1); *Enterobacter* sp. (1); *Erwinia* sp. (4); *Ewingella* sp. (1); *Klebsiella oxytoca* (2); *Klebsiella* sp. (1); *Kluyvera* sp. (1); *Pantoea agglomerans* (1); *Pantoea ananatis* (1); *Pantoea* sp. (2); *Pseudomonas* sp. (4); *Pseudomonas taetrolens* (1); *Raoultella terrigena* (1); *Serratia marcescens* (3); *Staphylococcus aureus* (3); *Staphylococcus* sp. (1); *Streptococcus agalactiae* (1); *Streptococcus* sp. (1)
		*Anoura geoffroyi*(7)	*Nectarivore*	*Citrobacter freundii* (1); *Enterobacter* sp. (5); *Hafnia alvei* (1); *Hafnia* sp. (2); *Klebsiella* sp. (1); *Kluyvera ascorbata* (1); *Kluyvera* sp. (1); *Pantoea ananatis* (1); *Pantoea* sp. (1); *Raoultella terrigena* (1); *Serratia* sp. (1); *Staphylococcus capitis* (1); *Staphylococcus epidermidis* (1); *Staphylococcus* sp. (1); *Stenotrophomonas* sp. (1); *Streptococcus agalactiae* (1)
		*Glossophaga soricina*(1)	*Nectarivore*	*-*
	**Subfamily Carolliinae**			
		*Carollia perspicillata*(8)	*Frugivore*	*Acinetobacter* sp. (1); *Bacillus* sp. (1); *Enterobacter* sp. (4); *Escherichia* sp. (1); *Ewingella* sp. (1); *Leclercia adecarboxylata* (2); *Pantoea* sp. (2); *Pseudomonas putida* (2); *Pseudomonas* sp. (1); *Raoultella terrigena* (1); *Serratia marcescens* (4); *Staphylococcus* sp. (1)
	**Subfamily Glyphonycterinae**			
		*Glyphonycteris sylvestris*(2)	*Insectivore*	*Serratia marcescens* (1); *Staphylococcus* sp. (1)
	**Subfamily Stenodermatinae**			
		*Artibeus fimbriatus*(9)	*Frugivore*	*Acinetobacter* sp. (1); *Enterobacter asburiae* (1); *Enterobacter cloacae* (2); *Enterobacter* sp. (5); *Erwinia* sp. (1); *Escherichia coli* (4); *Klebsiella* sp. (1); *Lactococcus* sp. (2); *Leclercia adecarboxylata* (1); *Raoultella ornithinolytica* (1); *Raoultella* sp. (1); *Raoultella terrigena* (2); *Serratia marcescens* (2)
		*Artibeus lituratus*(5)	*Frugivore*	*Bacillus megaterium* (1); *Citrobacter freundii* (1); *Enterobacter cloacae* (1); *Enterobacter* sp. (2); *Escherichia coli* (2); *Klebsiella oxytoca* (1); *Lactococcus lactis* (2); *Lactococcus* sp. (1); *Pantoea agglomerans* (1); *Pantoea* sp. (2); *Serratia marcescens* (2); *Serratia* sp. (1)
		*Artibeus obscurus*(8)	*Frugivore*	*Enterobacter aerogenes* (1); *Enterobacter ludwigii* (1); *Enterobacter* sp. (6); *Enterococcus* sp. (1); *Erwinia* sp. (1); *Escherichia coli* (6); *Escherichia* sp. (4); *Hafnia* sp. (1); *Klebsiella oxytoca* (2); *Klebsiella* sp. (1); *Raoultella planticola* (1); *Raoultella terrigena* (1); *Serratia marcescens* (4); *Serratia* sp. (1);Sp*hingobacterium* sp. (1)
		*Dermanura cinerea*(2)	*Frugivore*	*Citrobacter* sp. (1); *Enterobacter* sp. (3); *Klebsiella* sp. (1); *Stenotrophomonas maltophilia* (1)
		*Platyrrhinus lineatus*(1)	*Frugivore*	*Enterobacter* sp. (4); *Kluyvera ascorbata* (1)
		*Platyrrhinus recifinus*(1)	*Frugivore*	*Enterobacter* sp. (1); *Raoultella* sp. (1); *Serratia marcescens* (1)
		*Pygoderma bilabiatum*(2)	*Frugivore*	*Enterobacter cloacae* (2); *Leclercia adecarboxylata* (2); *Leclercia* sp. (1); *Pseudomonas* sp. (1); *Serratia marcescens* (2); *Stenotrophomonas* sp. (1)
		*Sturnira lilium*(8)	*Frugivore*	*Citrobacter freundii* (2); *Citrobacter* sp. (2); *Enterobacter* sp. (1); *Escherichia coli* (8); *Escherichia* sp. (5); *Klebsiella* sp. (2); *Kluyvera ascorbata* (1); *Kluyvera* sp. (1); *Pantoea* sp. (1); *Pseudomonas* sp. (2); *Serratia marcescens* (2)
		*Sturnira tildae*(5)	*Frugivore*	*Aeromonas* sp. (1); *Cedecea* sp. (1); *Citrobacter freundii* (1); *Citrobacter* sp. (1); *Enterobacter* sp. (2); *Escherichia coli* (1); *Escherichia* sp. (3); *Kluyvera* sp. (1); *Providencia alcalifaciens* (3); *Pseudomonas* sp. (2); *Streptococcus gallolyticus* (1)
		*Vampyressa pusilla*(1)	*Frugivore*	*Staphylococcus* sp. (1)
**Family Molossidae**				
	**Subfamily Molossinae**			
		*Cynomops abrasus*(1)	*Insectivore*	*Providencia rettgeri* (2); *Providencia* sp. (1)
		*Molossops neglectus*(1)	*Insectivore*	*Enterococcus* sp. (1); *Providencia rettgeri* (2)
		*Molossus currentium*(1)	*Insectivore*	*Lactococcus* sp. (1); *Proteus* sp. (1); *Proteus vulgaris* (1)
		*Molossus molossus*(4)	*Insectivore*	*Enterococcus faecalis* (2); *Enterococcus* sp. (2); *Escherichia coli* (3); *Hafnia alvei* (2); *Hafnia* sp. (2);*Klebsiella oxytoca* (2); *Lactococcus* sp. (1); *Staphylococcus* sp. (1);
		*Molossus rufus*(2)	*Insectivore*	*Escherichia albertii* (1); *Escherichia coli* (1); *Proteus vulgaris* (3); *Salmonella* sp. (1)
**Family Vespertilionidae**				
	**Subfamily Vespertilioninae**			
		*Eptesicus taddeii*(2)	*Insectivore*	*Enterococcus* sp. (1); *Escherichia coli* (1); *Escherichia* sp. (1); *Hafnia alvei* (1); *Providencia* sp. (2); *Serratia* sp. (1)
		*Lasiurus ebenus*(1)	*Insectivore*	*Acinetobacter* sp. (1); *Enterobacter asburiae* (1); *Enterobacter cloacae* (1); *Escherichia vulneris* (1); *Klebsiella* sp. (1); *Leclercia* sp. (1); *Pseudomonas aeruginosa* (1); *Staphylococcus* sp. (1)
		*Histiotus velatus*(3)	*Insectivore*	*Ewingella* sp. (1); *Hafnia alvei* (3); *Hafnia* sp. (3);Sp*hingobacterium* sp. (1)
	**Subfamily Myotinae**			
		*Myotis albescens*(1)	*Insectivore*	*Plesiomonas shigelloides* (1); *Plesiomonas* sp. (1)
		*Myotis nigricans*(6)	*Insectivore*	*Hafnia alvei* (2); *Lactococcus garvieae* (2); *Lactococcus lactis* (1); *Serratia marcescens* (1); *Serratia* sp. (2); *Staphylococcus hominis* (1); *Staphylococcus xylosus* (1)
		*Myotis riparius*(2)	*Insectivore*	*Enterococcus faecalis* (1); *Hafnia alvei* (2); *Raoultella* sp. (1); *Raoultella terrigena* (1); *Serratia marcescens* (4)
		*Myotis ruber*(2)	*Insectivore*	*Cedecea* sp. (1); *Enterococcus faecalis* (1); *Ewingella americana* (2); *Hafnia alvei* (1); *Lactococcus lactis* (1); *Pseudomonas* sp. (1); *Serratia marcescens* (1)

Isolates belong to four bacteria phyla, divided into 15 families. Proteobacteria was the most abundant phylum in all the dietary guilds, representing 87% of the total samples, followed by Firmicutes with 12%, and Actinobacteria and Bacteriodetes counting together 1% of the total identified samples. The family Enterobacteriaceae represented 73% of the samples, followed by Pseudomonadaceae, with 7%, and the other 20% are composed by small sums of the families Aeromonadaceae, Bacillaceae, Brucellaceae, Burkholderiaceae, Caulobacteraceae, Enterococcaceae, Lysobacteraceae, Microbacteriaceae, Micrococcaceae, Moraxellaceae, Neisseriaceae, Sphingobacteriaceae, Staphylococcaceae and Streptococcaceae. The phylum Actinobacteria, represented by *Arthrobacter* sp. and *Microbacterium* sp. was found only in the oral cavity, while the phylum Bacteroidetes is represented only by *Sphingobacterium* sp. in the rectal cavity.

**Fig 1:**
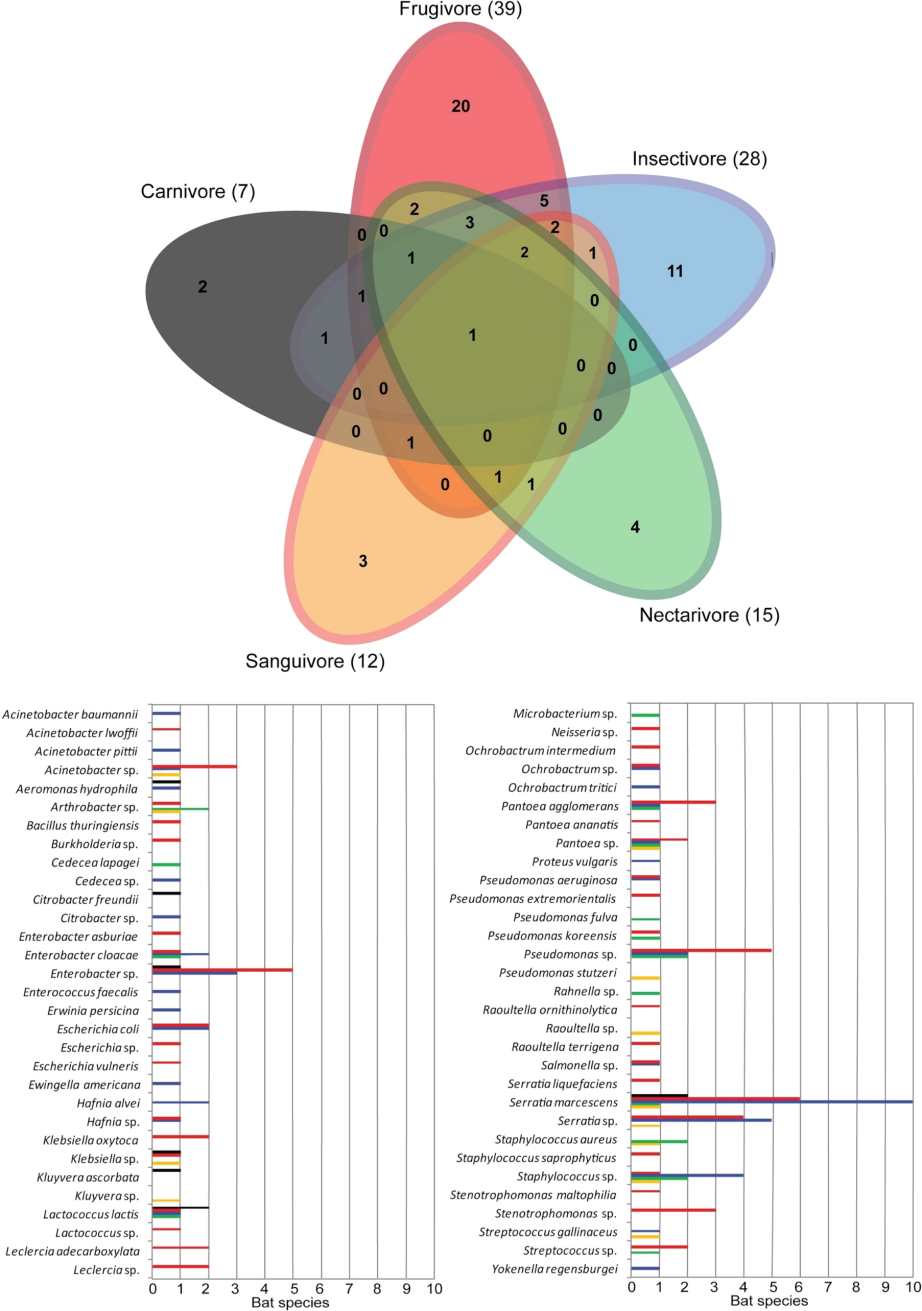
Venn-diagram showing the distribution of bacterial taxa from oral swabs of five dietary guilds of bats on Carlos Botelho State Park, São Paulo State. The number of taxa within each guild is represented in parenthesis. The abundance of each taxa on bat species is presented in the graph, and separated by dietary guilds.

**Fig 2:**
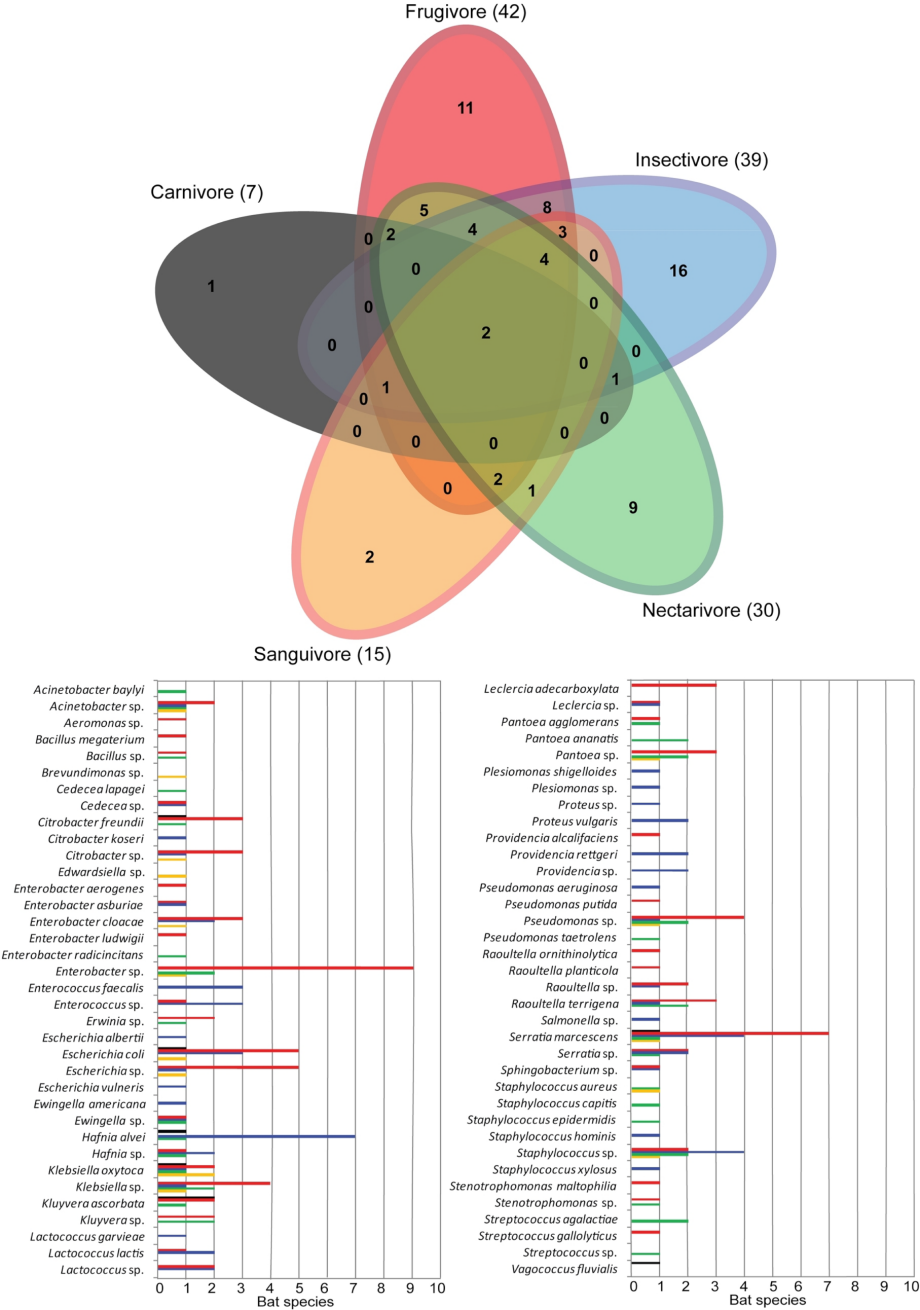
Venn-diagram showing the distribution of bacterial taxa from rectal swabs of five dietary guilds of bats on Carlos Botelho State Park, São Paulo State. The number of taxa within each guild is represented in parenthesis. The abundance of each taxa on bat species is presented in the graph, and separated by dietary guilds.

Sixty-two taxa of bacteria were identified in the oral cavity and 72 in rectal cavity of the bats. The Venn diagram analysis (Figs 1 and 2) indicates that the major proportion of the bacteria within the dietary guilds is shared between two or more guilds. The oral richness shared between guilds varies from 49% to 75%, whereas the rectal richness varies from 59% to 87% of bacteria taxa shared with at least one other guild. However, only the species *S*. *marcescens* is shared between all five guilds when the oral richness is analyzed alone, and only the species *K*. *oxytoca* and *S*. *marcescens* are shared between all the guilds when considered the rectal richness. Comparing the dietary guilds, higher richness was found on frugivores (58 taxa), followed by insectivores (50 taxa), nectarivores (37 taxa), sanguivores (21 taxa) and carnivores (11 taxa).

### Antibiotic sensitivity

Strains of one *A*. *baumannii*, 20 *E*. *coli*, 13 *K*. *oxytoca*, two *P*. *aeruginosa*, two *Salmonella* sp., 36 *S*. *marcescens*, two *S*. *maltophilia* and five *Stenotrophomonas* sp. were selected as the most potentially pathogenic isolates and tested for their susceptibility for antibiotics. The *A*. *baumannii* isolate was resistant only to ciprofloxacin, intermediate to ceftriaxone and sensible to all the other tested antibiotics. The two *P*. *aeruginosa* isolates were sensible to all the antibiotics tested. The two *Salmonella* sp. isolates exhibited different sensitivity, with one sensible to all the antibiotics tested, and the other resistant to the antibiotics ampicillin and cephalexin. Two *S*. *maltophilia* and five *Stenotrophomonas* sp. isolates also exhibited differences in sensitivity, with all the isolates resistant to the antibiotics ceftriaxone and imipenem, only one isolate sensible to the antibiotic gentamicin, and with variable sensitivity to the antibiotic ceftazidime (Table 3).

**Table 3:** Antibiotic-resistance patterns of *Acinetobacter baumannii*, *Pseudomonas aeruginosa*, *Salmonella* sp., *Stenotrophomonas maltophilia* and *Stenotrophomonas* sp. from swabs of bats on Carlos Botelho State Park, Brazil. The resistance patterns are classified as Sensitive (S), Intermediate (I) and Resistant (R). See Materials and Methods section for description of diet and antibiotics.

Bat species	Diet	Bacteria	Cavity	SUT	AMI	CAZ	CRO	CIP	CLO	DOX	GEN	IPM	NOR	AMC	AMP	CFL
*Molossus rufus*	INS	*Acinetobacter baumannii*	Oral	-	S	S	I	R	S	-	S	S	S	-	-	-
*Carollia perspicillata*	FRU	*Pseudomonas aeruginosa*	Oral	-	-	S	S	S	-	-	S	S	S	-	-	-
*Lasiurus ebenus*	INS	*Pseudomonas aeruginosa*	Oral	-	-	S	S	S	-	-	S	S	S	-	-	-
*Artibeus lituratus*	FRU	*Salmonella* sp.	Oral	-	S	S	S	S	S	S	S	S	-	S	R	R
*Molossus rufus*	INS	*Salmonella* sp.	Rectal	-	S	S	S	S	S	S	S	S	-	S	S	S
*Carollia perspicillata*	FRU	*Stenotrophomonas maltophilia*	Oral	S	-	S	R	S	-	-	R	R	S	-	-	-
*Dermanura cinerea*	FRU	*Stenotrophomonas maltophilia*	Rectal	S	-	S	R	S	-	-	R	R	S	-	-	-
*Artibeus fimbriatus*	FRU	*Stenotrophomonas* sp.	Oral	S	-	R	R	S	-	-	R	R	S	-	-	-
*Artibeus obscurus*	FRU	*Stenotrophomonas* sp.	Oral	S	-	R	R	S	-	-	R	R	S	-	-	-
*Pygoderma bilabiatum*	FRU	*Stenotrophomonas* sp.	Rectal	S	-	R	R	S	-	-	S	R	S	-	-	-
*Sturnira tildae*	FRU	*Stenotrophomonas* sp.	Oral	S	-	S	R	S	-	-	R	R	S	-	-	-
*Anoura geoffroyi*	NEC	*Stenotrophomonas* sp.	Rectal	S	-	S	R	S	-	-	R	R	S	-	-	-

The 20 *E*. *coli* isolates responses to the antibiotics tested were variable. Resistance to the antibiotics was absent for 16 of the isolates (80% of *E*. *coli* isolates), one isolate (5% of *E*. *coli* isolates) was resistant to ampicillin, one isolate (5% of *E*. *coli* isolates) was resistant to ampicillin and cephalexin, and two (10% of *E*. *coli* isolates) were resistant to amoxicillin-clavulanate, ampicillin and cephalexin (Table 4). From the 13 *K*. *oxytoca* isolates, seven (54% of *K*. *oxytoca* isolates) showed resistance to ampicillin, five (38% of *K*. *oxytoca* isolates) were intermediate to ampicillin, and one (8% of *K*. *oxytoca* isolates) was resistant to amoxicillin-clavulanate, ampicillin and cephalexin (Table 5). From the 36 *S*. *marcescens* isolates, 34 (95% of *S*. *marcescens* isolates) presented resistance to the antibiotics amoxicillin-clavulanate, ampicillin and cephalexin, and only two isolates (5% of *S*. *marcescens* isolates) were not resistant to amoxicillin-clavulanate and ampicillin (Table 6).

**Table 4:** Antibiotic-resistance patterns of *Escherichia coli* from swabs of bats on Carlos Botelho State Park, Brazil. The resistance patterns are classified as Sensitive (S), Intermediate (I) and Resistant (R). See Materials and Methods section for description of diet and antibiotics.

Bat species	Diet	Bacteria	Cavity	AMI	CAZ	CRO	CIP	CLO	DOX	GEN	IPM	AMC	AMP	CFL
*Trachops cirrhosus*	CAR	*Escherichia coli*	Rectal	S	S	S	S	S	S	S	S	S	S	S
*Artibeus fimbriatus*	FRU	*Escherichia coli*	Rectal	S	S	S	S	S	S	S	S	S	S	S
*Artibeus fimbriatus*	FRU	*Escherichia coli*	Rectal	S	S	S	S	S	S	S	S	S	S	I
*Artibeus lituratus*	FRU	*Escherichia coli*	Rectal	S	S	S	S	S	S	S	S	S	S	S
*Artibeus obscurus*	FRU	*Escherichia coli*	Oral	S	S	S	S	S	S	S	S	S	S	S
*Artibeus obscurus*	FRU	*Escherichia coli*	Rectal	S	S	S	S	S	S	S	S	S	S	S
*Artibeus obscurus*	FRU	*Escherichia coli*	Rectal	S	S	S	S	S	S	S	S	S	S	S
*Sturnira lilium*	FRU	*Escherichia coli*	Oral	S	S	S	S	S	S	S	S	S	S	S
*Sturnira lilium*	FRU	*Escherichia coli*	Rectal	S	S	S	S	S	S	S	S	S	S	S
*Sturnira lilium*	FRU	*Escherichia coli*	Rectal	S	S	S	S	S	S	S	S	S	S	S
*Sturnira lilium*	FRU	*Escherichia coli*	Rectal	S	S	S	S	S	S	S	S	S	S	S
*Sturnira lilium*	FRU	*Escherichia coli*	Rectal	S	S	S	S	S	S	S	S	S	S	S
*Sturnira tildae*	FRU	*Escherichia coli*	Rectal	S	S	S	S	S	S	S	S	S	S	S
*Desmodus rotundus*	SAN	*Escherichia coli*	Rectal	S	S	S	S	S	S	S	S	S	S	S
*Desmodus rotundus*	SAN	*Escherichia coli*	Rectal	S	S	S	S	S	S	S	S	S	R	S
*Eptesicus taddeii*	INS	*Escherichia coli*	Rectal	S	S	S	S	S	S	S	S	R	R	R
*Molossops neglectus*	INS	*Escherichia coli*	Oral	S	S	S	S	S	S	S	S	R	R	R
*Molossus molossus*	INS	*Escherichia coli*	Rectal	S	S	S	S	S	S	S	S	S	S	S
*Molossus rufus*	INS	*Escherichia coli*	Oral	S	S	S	S	S	S	S	S	S	R	R
*Molossus rufus*	INS	*Escherichia coli*	Rectal	S	S	S	S	S	S	S	S	S	S	S

**Table 5:** Antibiotic-resistance patterns of *Klebsiella oxytoca* from swabs of bats on Carlos Botelho State Park, Brazil. The resistance patterns are classified as Sensitive (S), Intermediate (I) and Resistant (R). See Materials and Methods section for description of diet and antibiotics.

Bat species	Diet	Bacteria	Cavity	AMI	CAZ	CRO	CIP	CLO	DOX	GEN	IPM	AMC	AMP	CFL
*Mimon bennetti*	CAR	*Klebsiella oxytoca*	Rectal	S	S	S	S	S	S	S	S	S	I	S
*Artibeus fimbriatus*	FRU	*Klebsiella oxytoca*	Oral	S	S	S	S	S	S	S	S	S	R	S
*Artibeus fimbriatus*	FRU	*Klebsiella oxytoca*	Oral	S	S	S	S	S	S	S	S	S	R	S
*Artibeus lituratus*	FRU	*Klebsiella oxytoca*	Oral	S	S	S	S	S	S	S	S	S	R	S
*Artibeus obscurus*	FRU	*Klebsiella oxytoca*	Rectal	S	S	S	S	S	S	S	S	R	R	R
*Platyrrhinus lineatus*	FRU	*Klebsiella oxytoca*	Oral	S	S	S	S	S	S	S	S	S	R	S
*Desmodus rotundus*	SAN	*Klebsiella oxytoca*	Rectal	S	S	S	S	S	S	S	S	S	I	S
*Desmodus rotundus*	SAN	*Klebsiella oxytoca*	Rectal	S	S	S	S	S	S	S	S	S	I	S
*Desmodus rotundus*	SAN	*Klebsiella oxytoca*	Rectal	S	S	S	S	S	S	S	S	S	I	S
*Desmodus rotundus*	SAN	*Klebsiella oxytoca*	Rectal	S	S	S	S	S	S	S	S	S	R	S
*Diphylla ecaudata*	SAN	*Klebsiella oxytoca*	Rectal	S	S	S	S	S	S	S	S	S	I	S
*Molossus molossus*	INS	*Klebsiella oxytoca*	Rectal	S	S	S	S	S	S	S	S	S	R	S
*Anoura caudifer*	NEC	*Klebsiella oxytoca*	Rectal	S	S	S	S	S	S	S	S	S	R	S

**Table 6:** Antibiotic-resistance patterns of *Serratia marcescens* from swabs of bats on Carlos Botelho State Park, Brazil. The resistance patterns are classified as Sensitive (S), Intermediate (I) and Resistant (R). See Materials and Methods section for description of diet and antibiotics.

Bat species	Diet	Bacteria	Cavity	AMI	CAZ	CRO	CIP	CLO	DOX	GEN	IPM	AMC	AMP	CFL
*Mimon bennetti*	CAR	*Serratia marcescens*	Oral	S	S	S	S	S	S	S	S	R	R	R
*Trachops cirrhosus*	CAR	*Serratia marcescens*	Oral	S	S	S	S	S	S	S	S	R	R	R
*Artibeus fimbriatus*	FRU	*Serratia marcescens*	Rectal	S	S	S	S	S	S	S	S	R	R	R
*Artibeus fimbriatus*	FRU	*Serratia marcescens*	Oral	S	S	S	S	S	S	S	S	R	R	R
*Artibeus lituratus*	FRU	*Serratia marcescens*	Rectal	S	S	S	S	S	S	S	S	R	R	R
*Artibeus obscurus*	FRU	*Serratia marcescens*	Rectal	S	S	S	S	I	S	S	S	R	R	R
*Artibeus obscurus*	FRU	*Serratia marcescens*	Oral	S	S	S	S	S	S	S	S	R	R	R
*Artibeus obscurus*	FRU	*Serratia marcescens*	Rectal	S	S	S	S	S	S	S	S	R	R	R
*Carollia perspicillata*	FRU	*Serratia marcescens*	Rectal	S	S	S	S	S	S	S	S	R	R	R
*Carollia perspicillata*	FRU	*Serratia marcescens*	Rectal	S	S	S	S	S	S	S	S	R	R	R
*Dermanura cinerea*	FRU	*Serratia marcescens*	Oral	S	S	S	S	I	S	S	S	R	R	R
*Platyrrhinus recifinus*	FRU	*Serratia marcescens*	Rectal	S	S	S	S	S	S	S	S	R	R	R
*Pygoderma bilabiatum*	FRU	*Serratia marcescens*	Rectal	S	S	S	S	S	S	S	S	R	R	R
*Pygoderma bilabiatum*	FRU	*Serratia marcescens*	Rectal	S	S	S	S	S	S	S	S	R	R	R
*Sturnira lilium*	FRU	*Serratia marcescens*	Rectal	S	S	S	S	S	S	S	S	R	R	R
*Glyphonycteris sylvestris*	INS	*Serratia marcescens*	Rectal	S	S	S	S	S	S	S	S	R	R	R
*Histiotus velatus*	INS	*Serratia marcescens*	Oral	S	S	S	S	S	S	S	S	R	R	R
*Lasiurus ebenus*	INS	*Serratia marcescens*	Oral	S	S	S	S	S	S	S	S	R	R	R
*Micronycteris microtis*	INS	*Serratia marcescens*	Oral	S	S	S	S	I	S	S	S	R	R	R
*Molossops neglectus*	INS	*Serratia marcescens*	Oral	S	S	S	S	S	S	S	S	I	S	R
*Molossus* cf. *currentium*	INS	*Serratia marcescens*	Oral	S	S	S	S	S	S	S	S	R	R	R
*Molossus molossus*	INS	*Serratia marcescens*	Oral	S	S	S	S	S	S	S	S	R	R	R
*Molossus molossus*	INS	*Serratia marcescens*	Oral	S	S	S	S	S	S	S	S	R	R	R
*Molossus rufus*	INS	*Serratia marcescens*	Oral	S	S	S	S	I	S	S	S	R	R	R
*Myotis nigricans*	INS	*Serratia marcescens*	Oral	S	S	S	S	S	S	S	S	R	R	R
*Myotis nigricans*	INS	*Serratia marcescens*	Oral	S	S	S	S	S	S	S	S	R	R	R
*Myotis nigricans*	INS	*Serratia marcescens*	Oral	S	S	S	S	S	S	S	S	R	R	R
*Myotis riparius*	INS	*Serratia marcescens*	Rectal	S	S	S	S	S	S	S	S	R	R	R
*Myotis ruber*	INS	*Serratia marcescens*	Rectal	S	S	S	S	S	S	S	S	R	R	R
*Myotis ruber*	INS	*Serratia marcescens*	Oral	S	S	S	S	S	S	S	S	R	R	R
*Anoura caudifer*	NEC	*Serratia marcescens*	Oral	S	S	S	S	S	S	S	S	R	R	R
*Anoura caudifer*	NEC	*Serratia marcescens*	Oral	S	S	S	S	S	S	S	S	R	R	R
*Anoura caudifer*	NEC	*Serratia marcescens*	Rectal	S	S	S	S	S	S	S	S	R	R	R
*Desmodus rotundus*	SAN	*Serratia marcescens*	Oral	S	S	S	S	S	S	S	S	R	R	R
*Desmodus rotundus*	SAN	*Serratia marcescens*	Oral	S	S	S	S	S	S	S	S	S	S	R
*Diphylla ecaudata*	SAN	*Serratia marcescens*	Rectal	S	S	S	S	S	S	S	S	R	R	R

## Discussion

### Bacteria richness

Gram-negative bacteria in the phylum Proteobacteria seems to be common in bat microbiota on studies based both on culture protocols and DNA sequencing, being isolated from oral and rectal cavities [8], intestine [9, 18] and saliva [10]. The phyla Actinobacteria, Bacteriodetes and Firmicutes were also previously reported as common on bats [10, 11, 18].

The mammalian gut microbiota diversity is related to the host diet, and should increase from animal-based diets to omnivorous to herbivore diets [29]. In our results, the frugivores microbiota was the most diverse among the five analyzed dietary guilds, and agrees to the mammalian gut microbiota theory. The less diverse microbiota in our survey was found in carnivores, which is also in agreement to the mammal microbiota theory. However, insectivores also showed high microbiota richness, and diverge from the expected, which could be explained by the inclusion of different alimentary items, rather than insects, on the diet of many species classified as insectivores. Species such as *Glyphonycteris sylvestris*, *Lampronycteris brachyotis*, *Micronycteris microtis* and *Myotis nigricans* analyzed in this study are reported to complement their diet with fruits and/or pollen [30–32], which could increase the general microbiota richness of the insectivore bats guild analyzed here.

Another possible explanation for the richness observed in the different bat guilds lies within the number of bats sampled for each guild, whereas the most diverse guilds are also the ones with more bat captures.Though most of the results are in agreement to other studies based on DNA sequencing, the general bacteria richness of bats from CBSP may be biased by the identification technique and the culture step. On the other hand, some bacteria genera, including pathogenic ones, are hard to speciate using DNA sequencing techniques [11], making comparisons even harder.

Some bacteria genera, such as *Arthrobacter*, *Burkholderia*, *Microbacterium*, *Neisseria* and *Rahnella* were found only in the oral cavity. *Arthrobacter* is composed by soil bacteria, and was also found on bats’ wing sacs, chin and axillae by other authors [33–35]; strains of *Arthrobacter* and *Rahnella* were identified as effective inhibitory antagonists of the growth of *Pseudogymnoascus destructans*, the fungus that causes white-nose syndrome, a letal bat disease [36]. *Burkholderia* and *Microbacterium* were previously found on bats’ saliva, urine, faeces, and intestine [9, 10]. *Neisseria* was previously found on bat saliva samples [10], and is closely related to mucosal and dental surfaces, being a consistent component of human oral microbiota and also found in different mammals [37]. The rectal cavity exclusive genus *Enterococcus* was also isolated from bats’ wings [35]. *Brevundimonas*, found only on the rectal cavity, was originally isolated from water and hospital-related material. This bacterium has been previously reported for marine mammals and is not common in bats [38, 39].

Bacteria genera observed within different dietary guilds were also divergent, with some exclusive occurrences. *Edwardsiella* was found only in sanguivores. This bacterium was previously isolated from bovine faeces and latter from cattle meat, wild mammals and birds [40, 41]. Thus, the occurrence of this bacterium only in this guild appears to be related to the feeding habit, which is based on blood from domestic and wild mammals and birds [42]. *Plesiomonas*, *Proteus* and *Yokenella* were identified only in insectivores. The genus *Proteus*, however, was also found in sanguivores and frugivores in other studies [8, 43]. The genus *Plesiomonas* is reported to be isolated from freshwater and surface water samples [44], and many species of insectivores are associated to these environments [45–47], where they forage and could be exposed to bacteria. *Yokenella* was previously isolated from the intestinal tracts of insects and faeces of insect-feeding animals, including bats [48, 49]; therefore it is probably related to this kind of diet. *Vagococcus*, here observed only in carnivores, has been recovered from animals, water, soil and human sources [50]. *S*.*marcescens* and *K*. *oxytoca* were found on all five dietary guilds. *S*.*marcescens* was reported in other studies and various dietary guilds, including frugivores [7], sanguivores [8, 43], and insectivores[11, 35]. *K*. *oxytoca* was previously reported in frugivores [7, 51] and insectivores[14, 52]; however, *K*. *oxytoca* was highly related to vespertilionid (insectivores) bats rather than to any other Australian mammal on previous studies [53].

### Antibiotic sensitivity

Generally, the resistance to antibiotics found on our samples was related to the intrinsic resistance of the tested species [54] and independent of dietary guilds of the bats. The species *P*. *aeruginosa* and *S*. *maltophilia* did not show any resistance besides their expected intrinsic resistance patterns. The species *A*. *baumannii* and *Stenotrophomonas* sp. showed resistance to the antibiotics ciprofloxacin and ceftazidime, respectively; those resistances are not intrinsic and could be acquired from both clinical or environmental antibiotic resistance genes sources, disseminated on the environment. *A*. *baumannii* is one of the most important pathogens in hospitals, and the development of multidrug-resistant strains has become of great concern for antibiotic therapies. Ciprofloxacin is a very potent antibiotic used as first line agaist *A*. *baumannii* infections [55–57] and previous studies have isolated high rates of ciprofloxacin resistant strains of *A*. *baumannii* [58–60]. The development of resistance on *A*. *baumannii* strains has been previously related to mutations in the quinolone resistance determining regions and efflux pump mechanisms [58, 59]. The only strain of *A*. *baumannii* was isolated from an insectivore bat that was found during the day on the floor of a Visitors Center on CBSP, and the possible contact of the bat with human leavings could have influenced on the acquiring of resistant strains. The *Stenotrophomonas* sp. resistant strains were found on frugivorous and nectarivorous and could outcome from the contact with water or fruits and even casual ingestion of insects [19–21].

Additionally, once the contact with anthropic and agricultural environments is one of the major sources of acquired resistance, the activity pattern and diet of carnivores, insectivores and sanguivores bats would make them more susceptible to exposure to antimicrobials [21, 61, 62]. Therefore, it could be expected that carnivore, insectivore and sanguivore bats would present a higher rate of antibiotic resistant strains, when compared to frugivores and nectarivores. However, this pattern is not clear when we analyse the results obtained for the antibiograms of the abundant bacteria species *E*. *coli*, *K*. *oxytoca* and *S*. *marcescens* to compare the dietary guilds. A larger number of samples and complementary analysis could help to better evaluate this question.

The tested *K*. *oxytoca* isolates presented only one resistant strain (5%) and none of the *S*. *marcescens* isolates presented any resistance besides the intrinsic ones. The small rates of resistant bacteria observed on CBSP in consistently different from those observed in other studies conducted on areas influenced by anthropic activities [17, 20, 63, 64]. A study conducted on Krakatau Islands found a great number of resistant bacteria on local bats and rats, which they correlated, in part, to anthropic influence on the local islands [20]. The antibiotic-resistance pattern found for *E*. *coli* isolates from Nigerian bats also showed a great number of resistant isolates; the resistance was attributed to the use of antibiotics on poultry feed or on poultry itself [17]. Analyzing all of our tested isolates, 71 out of 81 (87%) did not present any resistance besides the expected from the intrinsic pattern, which could be related to the effectiveness of CBSP on the conservation of the wildlife and environment present on the preserved area of the Park. Once some of the sampling sites were close to the Park limits and some Brazilian bats are know to forage on distances of 0.5 to 15 km [65–67], it seens that bats from CBSP prefer to forage on the pristine environments rather than anthropized surroundings. Moreover, the restriction of the contact to antibiotics would not lead to the decline of acquired resistances; therefore, it is reasonable to expect that resistance patterns on CBSP were always similar to the results presented here and no previous chronic exposures existed [68]. This result is in agreement to previous studies [22, 69], which reported a lack of human-acquired antibiotic resistance on environments with minimal anthropic influence and no chronic exposure to antibiotics.

Besides direct exposure to antibiotics, bacterial resistance can be originated through horizontally mobile elements such as conjugative plasmids, integrons and transposons [21]. Therefore, the low rate of resistance found on the Enterobacteriaceae from CBSP also suggests a small probability of the diffusion of acquired resistance on the Park. Many authors reported that bacteria from remote areas could work as sentinels and help to evaluate the impact of anthropic pressure on wildlife and the role of wild-species and natural environments on the process of resistance acquiring, which includes not only the exposure to antibiotics but also horizontal transference [21–23]. Our findings reinforce the need of monitoring antimicrobial resistance in wildlife from remote areas, appearing to be an effective tool to evaluate the environment responses to anthropic pressures. On this way, more efforts should be carried out on the Park to better evaluate local resistance patterns, the impact that the human activites of the surroundings on the Park environment and the role of wildlife as reservoirs of resistant bacteria.
